# Gynecomastia Outpatient Surgical Treatment in Children Without Closed-Suction Drain Placement: Is It Safe and Effective?

**DOI:** 10.3390/children12111571

**Published:** 2025-11-19

**Authors:** Carlos Delgado-Miguel, Ennio Fuentes, Pablo Aguado, Ricardo Díez

**Affiliations:** 1 Department of Pediatric Surgery, Fundación Jiménez Díaz University Hospital, 28040 Madrid, Spain; 2 Institute for Health Research IdiPAZ, La Paz University Hospital, 28046 Madrid, Spain

**Keywords:** gynecomastia, mastectomy, outpatient surgery, pediatric, drainless technique

## Abstract

**Introduction:** Idiopathic gynecomastia is a common diagnosis among adolescents. Closed-suction drain placement after breast resection is traditionally performed to prevent complications such as seroma or hematoma, although its effectiveness remains controversial. Drains are also associated with patient discomfort and may require prolonged wound care. The aim of the present study is to describe our experience with the outpatient surgical treatment of adolescent gynecomastia without closed-suction drain placement and to assess its short- and long-term outcomes. **Methods:** We conducted a retrospective single-center cohort study including male patients under 18 years of age operated on for idiopathic gynecomastia between 2019 and 2023. Demographic data, clinical features (grade of gynecomastia according to Simon’s classification before surgery), intraoperative variables, and postoperative outcomes were collected. Patients were followed in the outpatient setting, with assessment of early (<30 days) and late complications. **Results:** A total of 21 consecutive patients were included, with a median age of 14.5 years (range 13.6–17.4). Sixteen patients (76.2%) underwent bilateral mastectomies, and five (23.8%) underwent unilateral subtotal mastectomies. Gynecomastia grade was I in 6 cases (28.6%), II in 12 (57.1%), and III in 3 (14.3%). No intraoperative adverse events occurred. Compressive chest bandaging was performed without closed-suction drainage. All patients were discharged on the same day. Two patients (9.5%) developed mild seroma during follow-up, both resolving spontaneously without aspiration or surgery. **Conclusions:** Our preliminary findings suggest that outpatient gynecomastia surgery without closed-suction drains appears to be a safe and effective option in adolescents, minimizing discomfort and avoiding hospital admission. However, larger, multicenter comparative studies are warranted to confirm these results and to further evaluate patient satisfaction and long-term cosmetic outcomes.

## 1. Introduction

Gynecomastia is characterized by the benign enlargement of glandular breast tissue in males and represents the most frequent disorder of the male breast [1]. A wide range of etiologies has been described, including medications, hormonal imbalance, and neoplasms, although the condition is often idiopathic [2]. In adolescents, gynecomastia may significantly affect psychosocial well-being and body image [3,4]. When conservative management fails, surgical excision can restore chest contour and improve self-esteem.

Nipple-sparing subcutaneous mastectomy under general anesthesia remains the standard surgical approach [5]. Traditionally, closed-suction drains are placed to prevent postoperative fluid accumulation and to promote the closure of dead space [6,7]. However, their effectiveness in reducing hematoma or seroma formation remains controversial, with several studies—particularly in breast reduction and adult gynecomastia—reporting no significant benefit [8,9]. Furthermore, drains may increase patient discomfort, anxiety, and wound care burden, although some surgeons routinely perform outpatient procedures with short-term drains.

Adolescent gynecomastia differs from adult cases in several ways, including a higher glandular-to-fat ratio, firmer consistency, and greater skin elasticity, all of which influence surgical management and postoperative healing [10]. While liposuction alone may be sufficient in adults, adolescents often require direct glandular excision. Given the limited literature in pediatric patients and the potential to minimize invasiveness, the aim of this study was to evaluate the safety and feasibility of outpatient surgical treatment of gynecomastia without closed-suction drain placement, with attention to early and late complications.

## 2. Methods

We conducted a retrospective cohort study of consecutive male patients under 18 years of age who underwent surgery for idiopathic gynecomastia between 2019 and 2023 at our institution. Patients were excluded if they had secondary gynecomastia related to endocrine disorders, medication use, or tumors, a history of prior breast surgery, or incomplete clinical follow-up. Demographic variables, clinical features (grade of preoperative gynecomastia according to Simon’s classification), intraoperative data, postoperative outcomes, and morbidity within 30 days after surgery were analyzed. Comprehensive clinical and endocrine assessments were performed in all patients to exclude secondary causes of gynecomastia. A complete hormonal analysis was performed in all patients, and a preoperative breast ultrasound was also systematically conducted to rule out any underlying lesions or nodules. Grading of gynecomastia was performed according to the Simon classification system [11], which includes Grade I, minor enlargement without skin redundancy; Grade IIa, moderate enlargement without excess skin; Grade IIb, moderate enlargement with slight skin redundancy; and Grade III, severe enlargement with marked skin redundancy. Indications for surgical intervention included persistent gynecomastia after two years of conservative management or progressive glandular enlargement, psychological distress, or pain/discomfort despite conservative management.

**Figure 1:**
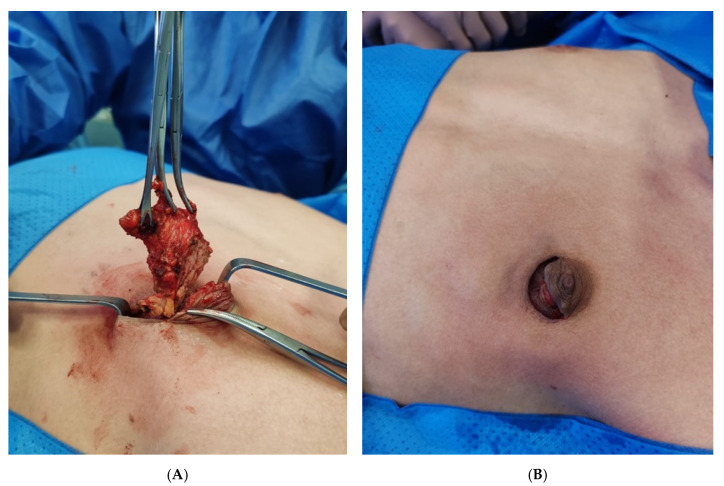
Intraoperative images of nipple-sparing subcutaneous mastectomy via infraareolar incision (**A**), and appearance after resection of glandular tissue, prior to skin closure of the incision (**B**), without placement of vacuum drains, reducing the number of incisions.

All procedures were performed under general anesthesia. Single-shot antibiotic intravenous prophylaxis (cefazolin, 1.0 g) was routinely administered at least 30 min before the start of the surgical procedure. Nipple-sparing subcutaneous mastectomy was performed using a semicircular periareolar approach, through a semicircular infra-areolar incision extending from the 3 o’clock to the 9 o’clock position, as described before [12]. Cooper’s ligaments dissection was performed reaching the pectoral fascia. Subsequently, the hypertrophic glandular tissue was removed gradually, preserving adequate tissue under the nipple-areola complex to ensure adequate perfusion and prevent further scarring and retraction into the pectoral fascia (Figure 1). Finally, hemostasis of the surgical site was checked, and skin closure was achieved using 4/0 absorbable sutures. Compressive chest bandaging was applied at the end of the procedure, and no closed-suction drains were placed. All patients were discharged on the day of surgery without the need for hospitalization. Postoperative antibiotic therapy was not administered. Postoperative pain management consisted of paracetamol, dosed according to patient weight. All the operations were performed by the same four surgeons with a similar approach. Resected specimens were weighed and sent for histopathological examination to confirm the diagnosis and rule out malignancy. The compressive elastic bandage was applied to the chest for 2 weeks postoperatively. Patients were instructed to avoid heavy physical activity and contact sports, to maintain the dressing daily, and to take paracetamol as needed for pain management.

Patients were routinely reviewed at 1 week and 4 weeks postoperatively for wound inspection and removal of the compressive dressing. Efficacy was evaluated through chest symmetry, contour, and functional recovery during clinical follow-up. Early complications such as hematoma, seroma, infection, or wound dehiscence were recorded within 30 days. Esthetic outcomes were assessed clinically by two independent surgeons, evaluating chest symmetry and contour, and through standardized pre- and postoperative photographs. Long-term complications (asymmetry, nipple retraction, hypertrophic scars, or recurrence) were assessed during follow-up visits beyond 6 months. The study protocol conformed to the guidelines of the 1975 Declaration of Helsinki and was approved by our institutional review board (number of approval: EO150-25). Child’s parents signed a written informed consent form, which included the publication of the images.

Statistical analysis was performed using Microsoft Excel (version 2010, Washington, DC, USA) and SPSS Statistics (version 23, IBM, Armonk, NY, USA). Categorical variables were expressed as frequencies (*n*) and percentages (%). Numerical variables were tested for normality using the Shapiro–Wilk and Kolmogorov–Smirnov tests to assess the normality of the distributions. Variables that followed a normal distribution were expressed as mean and standard deviation (SD), whereas those that did not follow a normal distribution were described as median and interquartile range (IQR).

## 3. Results

We included 21 patients with a median age of 14.5 years (range 13.6–17.4 years). Median weight was 68 kg (range 56–94 kg), and median height was 174 cm (range 162–184 cm). Grade of gynecomastia according to Simon’s classification was I in 6 cases (28.6%), II in 12 cases (57.1%) and III in 3 cases (14.3%). In the majority of patients, nipple-sparing subcutaneous mastectomy was performed bilaterally in 16 cases (76.2%), while in the remaining 5 patients the procedure was performed unilaterally (3 on the right side and 2 on the left). No intraoperative complications occurred. Compressive chest bandaging was performed, without closed-suction drain placement. The average surgery duration was 71 min (SD ± 16 min), and hemostasis was achieved using careful electrocoagulation of glandular pedicles with intraoperative bleeding review. All cases were discharged on the same day of surgery, with no need for hospitalization. No postoperative antibiotics were given. All patients received postoperative pain management with paracetamol, dosed based on their weight. Resected tissue had a median weigh of 135 g (range 94–154 g). All histological analyses confirmed the diagnosis of gynecomastia, with none showing evidence of malignancy. Table 1 summarizes the baseline demographic and clinical characteristics of the study population.

**Figure 2:**
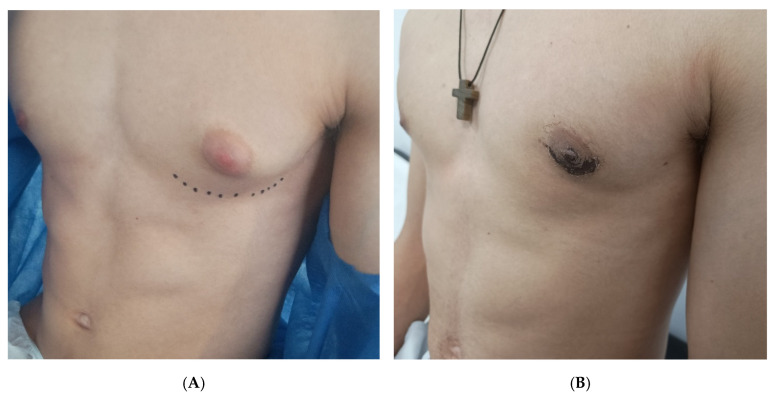
Left unilateral grade I gynecomastia in a 16-year-old patient. Preoperative appearance with lower breast margin marked with dotted line (**A**) and 10 days after surgery (**B**), where a small scab is observed over the incision, with no signs of hematoma or seroma.

**Figure 3:**
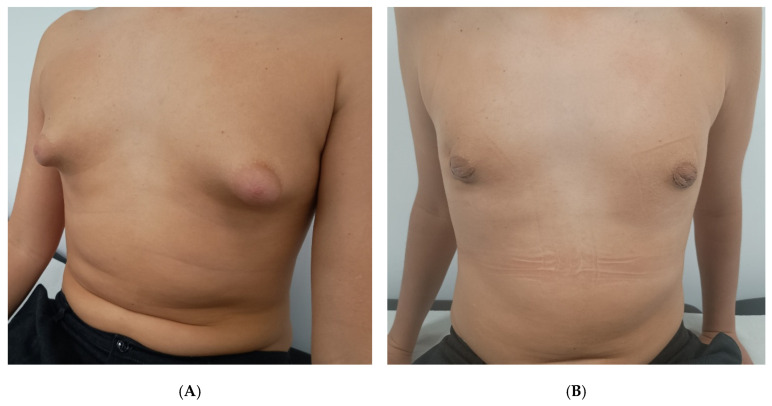
Bilateral grade II gynecomastia in a 14-year-old patient. Preoperative appearance (**A**) and 2 weeks after surgery (**B**), with very good esthetic results.

During the early postoperative period (within 30 days), two patients (9.5%; 95% CI, 1.2–30.4%) developed a mild seroma, presenting as localized swelling confirmed clinically. None required aspiration or further intervention; both resolved spontaneously within 10 days with continued compression and observation. No hematomas, wound infections, or dehiscence were identified. At long-term follow-up (median 28 months, range 15–46 months), no cases of recurrent gynecomastia, nipple–areola necrosis, or hypertrophic scarring were observed. All patients achieved satisfactory contour retraction and symmetry as evaluated clinically. Figure 2 shows a representative case of unilateral gynecomastia with preoperative and postoperative views, and Figure 3 demonstrates a bilateral case illustrating favorable skin adaptation and esthetic outcome.

## 4. Discussion

In this study we present our initial results of outpatient surgical treatment of gynecomastia in pediatric patients without closed-suction drain placement. Gynecomastia is the most common disorder of the male breast and can manifest unilaterally or bilaterally [1]. Reported prevalence rates vary between 36% and 65% in adults and 4% to 40% in adolescents, depending on the study population and criteria used for diagnosis [13]. In adolescents most cases are idiopathic, although can potentially be associated with endocrinological and urological diseases, and therefore, complementary studies should be performed to rule out alterations at these areas [1]. Independently of the underlying causes, the development of female-looking breasts can profoundly affect patients’ psychological state and general well-being [14]. Most adolescent gynecomastia is resolved spontaneously in 2 years, but persistent gynecomastia could have a negative influence on psychoemotional development on adolescence [15]. This distress is often the primary motivation for seeking surgical intervention in most patients [16]. Adolescents, who are navigating the challenges of identity formation and social integration, can be especially vulnerable to body image concerns like gynecomastia, and this psychological stress may indicate a need for surgical intervention [17,18].

Subcutaneous mastectomy with nipple preservation and liposuction, either individually or in combination, have formed the cornerstone of surgical approaches [2,12]. Complications following surgical management of gynecomastia may include hematoma, seroma, infection, incomplete resection, asymmetry, alterations in sensation, and contour irregularities [2]. Hematoma and seroma are the most frequent acute postoperative complications, and they entail an important comorbidity that on many occasions may require surgical reintervention to resolve them. Surgical practice often deems drains essential for eliminating dead space and evacuating effluent, in order to prevent the accumulation of collections in surgical area, by allowing the fluid to drain out. Nonetheless, closed-suction drains come with their own set of potential drawbacks, including heightened patient discomfort and anxiety, as well as additional expenses associated with drain site maintenance and clinic visits. In addition, they are frequently colonized by bacteria, which leads many practitioners to prescribe systemic antibiotics until drain removal [19]. All this explains why the placement of drains presents different advantages and disadvantages and is currently a controversial subject.

However, there is little evidence reported in children or adolescents, as most studies include adult patients. Arrowsmith et al. reported a similar rate of postoperative hematoma between patients with drain placement after breast reduction and those without drainage [20]. Chao et al. also analyzed the usefulness of drainage in adult patients with gynecomastia and found that drains did not reduce the rate of overall fluid collections or hematomas after subcutaneous mastectomy, although they significantly decreased the rate of seroma requiring drainage in breasts in which a drain was placed [10]. In our experience, avoidance of drains in children may even reduce hospital stays secondary to increased patient comfort, which agrees with the results of Corion et al. in adult patients [21]. Not placing a drain avoids, on the one hand, discomfort during the time that the drain persists, and on the other hand, it also avoids the anxiety and discomfort related to the removal of the drain, which in some anxious patients may require sedation to achieve adequate removal of the drain [10,21]. Alternatives to drains, such as fibrin sealants, have been investigated, appearing effective in promoting tissue adherence and reducing postoperative drainage after mastectomy, although again, experience in children is nil [22].

In our study, the semicircular-periareolar inferior incision proved to be a practical approach in all grades of ginecomastia. Even in severe cases of grade III gynecomastia, concentric skin excision with nipple reposition was not necessary, due to the adequate skin elasticity of the breasts. In contrast to other authors, the hospital stay was zero days, as all patients were discharged the same day of surgery. This avoids uncomfortable hospital stays due to the presence of the drain, as reported by Fisher et al. who operated on 37 adolescents with a mean inpatient stay of 4.5 days (median 4, range 3–7 days) [23]. Postoperative complication rate in our series was 9.5% due to seroma in two cases, which did not require surgical treatment, as they resolved with compressive bandaging during the following weeks. Other series of gynecomastia revealed complication rates ranging from 15.5 to 41% [24,25]. In previous studies, overweight was considered a risk factor for complications, but the two seromas observed in our study did not occur in overweight patients [26]. We routinely advised the prolonged use of a compressive dressing with an elastic bandage for 2 weeks during the postoperative course, but in one of the cases, she did not follow these recommendations and only maintained the compressive dressing for 48 h, subsequently enhancing the occurrence of seroma. Seroma is defined as a collection of fluid under the surface of the adipocutaneous flap, related to the amount of adipose tissue damage during the surgical procedure [27]. It occurs more frequently in extensive procedures compared to minor surgical procedures and is related to the amount and volume of resected tissue. However, as fluid is usually collected in the late postoperative period, it is very important to achieve adequate compression of the area during the first few weeks. Closed-suction drains are usually removed in the first 3–4 days, and therefore sometimes does not prevent seroma formation later on. This may explain the low postoperative seroma rate observed in our series. Long-term follow-up (median 28 months) confirmed the absence of recurrent gynecomastia or contour irregularities. This extended observation strengthens our findings, as late complications such as nipple retraction, hypertrophic scarring, or residual deformities may manifest months after surgery.

Given the absence of a universally accepted standard of care, some experts suggest that surgeons carefully evaluate the need for drain placement and engage in discussions with patients undergoing nipple-sparing subcutaneous mastectomy for gynecomastia regarding the associated costs, risks, and benefits of using drains [10]. Our results align with previous reports showing that drain omission does not increase the risk of hematoma or seroma, while improving patient comfort, and therefore, surgical treatment of gynecomastia without a closed-suction drain can be considered a safe treatment modality across adolescents [10,20]. On the one hand it reduces the discomfort associated with drainage, and on the other hand it avoids the need for hospital admission, allowing treatment to be performed as an outpatient procedure. The present findings have practical implications for pediatric surgical practice, suggesting that drainless outpatient gynecomastia surgery may safely reduce hospitalization and discomfort while maintaining esthetic outcomes. Theoretically, these results support the growing trend toward minimally invasive, patient-centered care in pediatric plastic surgery.

Our study has several important limitations to note, mainly stemming from its single-center design and descriptive retrospective analysis. As we did not place drainage in any patient, it makes it impossible to establish two groups for a potential comparison. Another notable limitation in our study is the scarcity of patients necessitating skin excision, likely due to the favorable elasticity observed in the adolescent demographic. It would be valuable to explore a distinct cohort inclusive of a broader range of skin types to evaluate the impact of skin tightening procedures on the incidence of postoperative fluid collections without closed-suction drain placement. Multicenter studies with larger numbers of patients could be helpful in addressing this limitation. Another risk factor for the development of complications such as postoperative hematomas is adequate hemostasis at the surgical site, which can vary among surgeons. Our series includes patients operated on by 4 surgeons, of which 2 usually participate in each intervention, each one performing the mastectomy on one side, so although stratified analysis by operating surgeon could provide additional insights, it was not feasible in our current study. An additional study limitation was our short 30-day follow-up, which limits the evaluation of medium- or long-term complications. However, the development of seroma or hematoma is very rare beyond the first 30 days, so these complication rates are unlikely to be underestimated. Future research should include randomized controlled trials comparing drain versus no-drain techniques, incorporating objective measures such as validated esthetic satisfaction scales, quality-of-life questionnaires, and standardized photographic assessments to better quantify outcomes and patient perception.

## 5. Conclusions

This retrospective series suggests that outpatient nipple-sparing subcutaneous mastectomy without drains is a feasible and apparently safe option for adolescent gynecomastia when performed by experienced surgeons with appropriate selection criteria. The procedure was associated with low morbidity, rapid recovery, and satisfactory esthetic results during long-term follow-up. However, given the limited sample size and absence of a comparison group, these findings should be considered preliminary. Larger, preferably multicenter prospective studies—including patient-reported outcomes and comparative analyses with drain-assisted surgery—are warranted to confirm the safety, efficacy, and patient comfort associated with this simplified ambulatory approach.

## Figures and Tables

**Table 1:** Demographic and clinical characteristics of the study population.

Variable	Median (Range) or n (%)
Age (years)	14.5 (13.6–17.4)
Weight (kg)	68 (56–94)
Height (cm)	174 (162–184)
Body Mass Index (kg/m^2^)	24.5 (23.1–26.2)
Simon Grade I	6 (28.6%)
Simon Grade II	12 (57.1%)
Simon Grade III	3 (14.3%)
Bilateral surgery	16 (76.2%)
Unilateral surgery	5 (23.8%)
Mean resection weight (per breast, g)	135 ± 22
Outpatient discharge	21 (100%)

## Data Availability

The data are available upon proper request.
